# High On-Aspirin Platelet Reactivity and Clinical Outcome in Patients With Stable Coronary Artery Disease: Results From ASCET (Aspirin Nonresponsiveness and Clopidogrel Endpoint Trial)

**DOI:** 10.1161/JAHA.112.000703

**Published:** 2012-06-22

**Authors:** Alf-Åge R. Pettersen, Ingebjørg Seljeflot, Michael Abdelnoor, Harald Arnesen

**Affiliations:** Center for Clinical Heart Research, Oslo University Hospital, Ullevaal, Norway (A.-Å.R.P., I.S., H.A.); Department of Cardiology, Oslo University Hospital, Ullevaal, Norway (A.-Å.R.P., I.S., H.A.); Section for Epidemiology and Statistics, Oslo University Hospital, Ullevaal, Norway (M.A.); Faculty of Medicine, University of Oslo, Oslo, Norway (I.S., H.A.)

**Keywords:** antiplatelet therapy, aspirin, clopidogrel, residual platelet reactivity, angina, stable

## Abstract

**Background:**

Patients with stable coronary artery disease on single-antiplatelet therapy with aspirin are still at risk for atherothrombotic events, and high on-aspirin residual platelet reactivity (RPR) has been suggested as a risk factor.

**Methods and Results:**

In this randomized trial, the association between platelet function determined by the PFA100 platelet function analyzer system (Siemens Healthcare Diagnostics, Germany) and clinical outcome in 1001 patients, all on single-antiplatelet therapy with aspirin (160 mg/d) was studied. Patients were randomized to continue with aspirin 160 mg/d or change to clopidogrel 75 mg/d. A composite end point of death, myocardial infarction, ischemic stroke, and unstable angina was used. At 2-year follow-up, 106 primary end points were registered. The prevalence of high RPR was 25.9%. High on-aspirin RPR did not significantly influence the primary end point in the aspirin group (13.3% versus 9.9%, *P*=0.31). However, in post hoc analysis, patients with von Willebrand factor levels or platelet count below median values and high on-aspirin RPR had a statistically significant higher end point rate than that of patients with low RPR (20% versus 7.5%, *P*=0.014, and 18.2% versus 10.8%, *P*=0.039, respectively). The composite end point rate in patients with high on-aspirin RPR treated with clopidogrel was not different from that of patients treated with aspirin (7.6% versus 13.3%, *P*=0.16).

**Conclusions:**

In stable, aspirin-treated patients with coronary artery disease, high on-aspirin RPR did not relate to clinical outcome and did not identify a group responsive to clopidogrel. Post hoc subgroup analysis raised the possibility that high on-aspirin RPR might be predictive in patients with low von Willebrand factor or platelet count, but these findings will require confirmation in future studies.

**Clinical Trial Registration:**

URL: http://www.clinicaltrials.gov Unique identifier: NCT00222261. **(*J Am Heart Assoc*. 2012;1:e000703 doi: 10.1161/JAHA.112.000703.)**

## Introduction

Despite the well-documented efficacy of aspirin in reducing myocardial infarction, stroke, and death, some patients on aspirin experience new cardiovascular events. This has led to the introduction of the concepts of aspirin nonresponsiveness, aspirin resistance, or high on-treatment residual platelet reactivity (RPR).^1,2^

Several reports have shown, by laboratory testing, insufficient platelet inhibition in 5% to 40% of aspirin-treated patients. Different laboratory methods have been used in the evaluation of response to aspirin, such as platelet reactivity index, platelet aggregate ratio, platelet aggregation (optical or by impedance), the PFA100 platelet function analyzer method (Siemens Healthcare Diagnostics, Germany), and lately the VerifyNow Aspirin method (Accumetrics, San Diego, CA).^3^

Different mechanisms have been discussed as potential explanations of the laboratory phenomenon of aspirin nonresponsiveness.^1,4–6^ Several studies have documented a substantial inhibition of the production of thromboxane A_2_ in aspirin-treated patients with high RPR, and thus the term *aspirin resistance* seems inappropriate.^7^

The clinical relevance of high on-aspirin RPR has been addressed in many trials.^8–14^ Nevertheless, current guidelines do not recommend routine use of platelet function tests in aspirin-treated patients.^1,15,16^

New antiplatelet drugs for long-term treatment of patients with coronary artery disease (CAD) have become available for clinical use.^2^ Clopidogrel has been widely used for the past decade in combination with aspirin in high-risk patients, often for a time-limited period. Clopidogrel also has been used in monotherapy as an antiplatelet agent in patients with contraindications to aspirin.^17^ Both drugs have shown large variations in the frequency of on-treatment RPR.^18–21^ It is not known whether patients with high on-aspirin RPR are better protected with clopidogrel.

The aim of the present study was to investigate the influence of high on-aspirin RPR on clinical outcome after 2 years in patients with documented CAD. The hypothesis was that high on-aspirin RPR as measured with the PFA100 method would translate into an increased rate of clinical end points after 2 years. In addition, we hypothesized that patients with high on-aspirin RPR would benefit from clopidogrel treatment.

## Methods

### Study Design

The ASpirin nonresponsiveness and Clopidogrel Endpoint Trial (ASCET) is a single-center, randomized open trial (double blinded for the results of platelet function tests), investigating the clinical outcome over a minimum period of 2 years in aspirin-treated, stable CAD patients as related to their RPR. Patients (n=1001) were randomized to either continue aspirin 160 mg/d or change to clopidogrel 75 mg/d after having given written informed consent in accordance with the recommendation of the revised Declaration of Helsinki. Randomization was undertaken by using consecutively numbered nontranslucent envelopes with computerized random allocation to the treatment groups. The clinical outcome was related to the patient's response to aspirin at baseline, assessed by the PFA100 method. Compliance to aspirin therapy was assessed by determination of serum thromboxane B_2_ (TxB_2_) levels and by written questionnaires. Follow-up visits were scheduled after 1, 12, and 24 months.

The study was approved by the local ethics committee. The details of the design have been published previously.^22^ The ASCET study is registered at http://www.clinicaltrials.gov (identification No. NCT00222261).

### Study Patients

The study included clinically stable patients of both sexes who were 18 to 80 years of age, had angiographically documented CAD, and were on long-term single-antiplatelet therapy with aspirin (160 mg/d) at randomization. Patients were not included as long as there still was an indication for dual-antiplatelet or warfarin treatment or if there were contraindications to any of the study drugs. Pregnant or breastfeeding women and patients with psychiatric disease or alcohol or drug abuse that could reduce patient compliance were also not included. Baseline characteristics of the study population are presented in Table 1.

**Table 1.** Baseline Characteristics of the Total Study Population (n=1001)

Age, y, mean (range)	62.4 (36–81)

Sex, female, n (%)	218 (21.8)

Race, white, n (%)	969 (96.8)

High RPR (PFA100), n (%)	259 (25.9)

Cardiovascular risk factors, n (%)	

Current smoking	204 (20.4)

Hypertension	555 (55.4)

Diabetes mellitus	200 (20.0)

Previous myocardial infarction	437 (43.7)

Intermittent claudication	54 (5.4)

Previous percutaneous coronary intervention	731 (73.0)

Previous coronary artery bypass grafting	185 (18.5)

Systolic blood pressure, mm Hg, mean±SD	139±19

Diastolic blood pressure, mm Hg, mean±SD	82±10

Body mass index, kg/m^2^, median (25th, 75th percentiles)	27.1 (24.8, 29.6)

Total cholesterol, mmol/L, mean±SD	4.55±0.98

Low-density lipoprotein cholesterol, mmol/L, mean±SD	2.53±0.83

High-density lipoprotein cholesterol, mmol/L, mean±SD	1.33±0.41

Triglycerides, mmol/L, median (25th, 75th percentiles)	1.31 (0.93, 1.85)

Platelet count, ×10^9^/L, median (25th, 75th percentiles)	227 (195, 264)

Mean platelet volume, fL, mean±SD	10.87±0.93

C-reactive protein, mg/L, median (25th, 75th percentiles)	1.90 (0.90, 3.30)

vWF, %, median (25th, 75th percentiles)	105 (82, 133)

TxB_2_, ng/mL, mean (range)	2.7 (0.00–21.00)

Medication, %	

Statins	98.3

β-Blockers	75.8

Calcium channel blockers	25.6

Angiotensin-converting enzyme inhibitors	26.4

Angiotensin receptor blockers	24.0

Proton pump inhibitors	11.0

Values are given as n (%), mean±SD, mean (range), median (25th, 75th percentiles), or percentage, as indicated.

RPR is defined as residual platelet reactivity assessed by PFA100; hypertension, previously diagnosed hypertension and/or currently treated hypertension; current smoking, regular tobacco smoking or <3 months since smoking cessation; and diabetes mellitus, previously diagnosed diabetes or fasting glucose ≥7 mmol/L.

### Study Medications

Tablets of aspirin (Albyl-E; Nycomed-Pharma, Norway) 160 mg and tablets of clopidogrel (Plavix; Sanofi Winthrop, Sweden, and Bristol-Myers Squibb SNC, Paris, France) 75 mg were used. The Albyl-E tablets were provided by Nycomed-Pharma. Because of a resolution of Rikstrygdeverket, Norway, the Plavix tablets were covered by the Act of National Insurance Administration. The hospital pharmacy stored and delivered the study drugs. Patients randomized to clopidogrel 75 mg/d were not given loading doses. The treatment was initiated on the day of randomization. All other medications were given according to current guidelines.

### Laboratory Methods

All blood samples were drawn between 8:00 and 10:30 am in fasting condition ≈24 hours after the most recent intake of medication. Routine analyses were performed with conventional laboratory methods. Citrated whole blood (sodium citrate [0.129 μmol/L in dilution 1:10]) was used for platelet function testing, which was carried out within 2 hours after blood sampling.

The PFA100 system (Siemens Healthcare Diagnostics, Germany) simulates platelet-based primary hemostasis in vitro. A syringe aspirates citrated whole blood under steady-flow conditions through a small aperture cut into a membrane in the test cartridge. The membrane in the cartridge used is coated with type I collagen and epinephrine. The time necessary for the occlusion of the aperture is defined as closure time, which is indicative of platelet function in whole blood.

To define the cutoff value for high RPR, we tested 200 CAD patients not on antiplatelet therapy (from the warfarin group of the Warfarin, Aspirin, Reinfarction Study [WARIS II]), and the 95th percentile in this group was used, giving a cutoff value of 196 seconds. ^8^ The term *high RPR* as defined by the PFA100 method has been used throughout in accordance to the study protocol.^22^

Whole blood without anticoagulants was collected and kept at 37°C for 1 hour before centrifugation at 2500×*g* for 10 minutes for serum TxB_2_ determination (Amersham Thromboxane B_2_ Biotrak Assay, GE Healthcare, Buckinghamshire, UK). Von Willebrand factor (vWF) was measured in citrated plasma with a commercial ELISA method (Asserachrom vWF Ag, Stago Diagnostica, Asnieres, France).

### Clinical Endpoints

The primary end point includes the first event of the composite of unstable angina with ECG changes or levels of cardiac markers not to be classified as a myocardial infarction, nonhemorrhagic stroke, myocardial infarction, or death. In all patients who were unable to attend the final visit, the clinical end points were recorded on request. Evaluation of end points was performed by an end point committee without access to the treatment code. Internationally accepted diagnostic criteria were used. A random selection of 50 (5%) of the completed Case Record Forms were monitored and approved by an independent consultant.

### Bleeding Classification

Major bleeding was defined as bleeding requiring transfusion of blood or surgical intervention. Intracranical bleeding was always classified as major bleeding. Minor bleeding was defined as bleeding not requiring transfusion or surgical intervention, including subcutaneous bruising, minor hematomas, and oozing from puncture sites or gums.

### Adverse Events

All adverse events for either of the study drugs were recorded throughout the study period. If considered necessary, the study drug was terminated. In the case of any serious event, the National Drug Authority was notified.

### Statistical Analysis

The observation time was a minimum of 2 years per patient. The number of patients needed to obtain a 40% reduction in the composite end point in patients with low on-aspirin RPR as compared to patients with high on-aspirin RPR (from 32% to 18%), provided that 30% had high RPR, was calculated to be 500, with type 1 error of 5% and 80% power. An additional 500 patients were included for the clopidogrel treatment.

Continuous variables are presented as mean±SD or median (25^th^, 75th percentiles) in skewed variables. Categorical variables are presented as numbers or percentages, as appropriate. Group comparisons were performed by Student unpaired *t* tests or Mann-Whitney *U* tests when appropriate for continuous variables and by the χ^2^ test or Fisher exact test for categorical variables. The analysis of end points was performed by the intention-to-treat principle, and rate ratios were calculated by using follow-up time as denominator in a 2×2 table (person-years model).^23^

For the group continuing on aspirin, patients with high RPR, as compared to patients with low RPR, were analyzed with regard to composite end points. This association was quantified by the crude odds ratio (OR) with 95% confidence interval (CI). Stratification analyses based on differences between the groups were performed. The Breslow-Day test of heterogeneity was used to pinpoint effect modification before quantifying potential confounders by the Mantel-Haenszel method. Because none of the tested variables appeared to be confounders, no adjustments were performed. The log-likelihood ratio test was performed to compare the model with and without interactions. Estimation of the OR for the different levels of interaction variables was done by using the variance-covariance matrix to estimate the correct variance of the OR.^24^ In the post hoc analyses, adjustments for relevant covariates (differences between groups at the level of *P*<0.20) were performed by logistic regression analyses. The Strengthening the Reporting of Observational Studies in Epidemiology (STROBE) guidelines were followed.^25^ A 2-tailed *P* value <0.05 was considered statistically significant. SPSS version 18.0 (SPSS Inc., IL, USA), Open Epi version 2.3.1 (AG Dean, KM Sullivan and MM Soe), Epiinfo version 3.5.1 (CDC, Atlanta, GA, USA), and Stata version 11 (StataCorp, College Station, TX, USA), were used.

### Administrative Matters

The ASCET study had a Steering Committee for monitoring the study's progress and for quality evaluation. An independent Data Safety and Monitoring Board was allowed access to the database during the study to assess data quality and evaluate the number of adverse events. All end points and serious adverse events were ultimately evaluated by the Data Safety and Monitoring Board before study closure.

## Results

**Figure 1.**
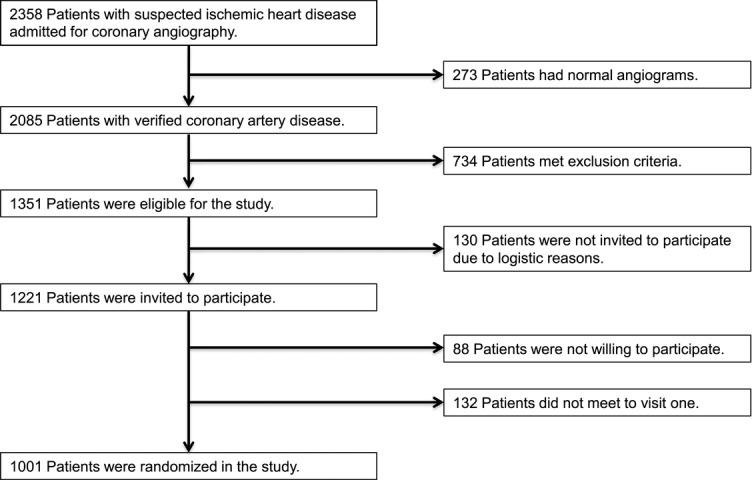
Selection and enrollment of study patients.

During the enrollment period (March 2003 to July 2008), 2358 patients undergoing coronary angiography because of clinical symptoms of CAD were screened for enrollment (Figure 1). A total of 1001 patients were enrolled in the study after consideration of inclusion and exclusion criteria, logistics, and patient consent. All patients were followed up for 2 years, and the study was completed in July 2010. Baseline characteristics of the total population are given in Table 1. The number of patients randomized to continue on aspirin was 502, and 499 were randomized to clopidogrel. The 2 randomized groups were well balanced with regard to all baseline characteristics, and there were no differences in the frequency of high on-aspirin RPR between the groups. The number of patients discontinuing study medication without having reached an end point was 95 (9.5%). This was mainly due to a new indication for dual-antiplatelet treatment (Table 2).

**Table 2.** Discontinuation of Study Drugs According to the Randomized Groups

	Aspirin (n = 502)	Clopidogrel (n = 499)	P*
Discontinuation† of study drug	32	63	0.001

Change to dual-antiplatelet treatment‡	23	23	0.98

Change to warfarin	5	10	0.19

Dyspepsia, peptic ulcer	3	5	0.47

Diarrhea	0	4	0.04

Rash	0	5	0.03

Other	1	16¶	<0.001

Values are given as numbers of patients.

* *P* for differences between groups.

† Discontinuation defined as being off the randomized principle for >28 days.

‡ Patients undergoing new percutaneous coronary intervention with stent implantation without reaching an end point.

¶ Includes dizziness (n=3), general discomfort (n=3), and discontinuation due to change of medication beyond the study protocol.

The total number of primary end points recorded was 106 (10.6%), which was the first event of unstable angina (n=33 [3.3%]), myocardial infarction (n=36 [3.6%]), ischemic stroke (n=28 [2.8%]), or death (n=9 [0.9%]). No difference in end point rates between the randomized groups was observed (54 of 502 on aspirin [10.8%] and 52 of 499 on clopidogrel [10.4%], *P*=0.87).

### RPR in the Total Study Population

In the total population, the frequency of high RPR on aspirin treatment evaluated by the PFA100 method at randomization was 25.9%. The mean level of serum TxB_2_ was 2.7 ng/mL (range, 0 to 21.0 ng/mL) at randomization, independent of on-aspirin RPR.

### High On-Aspirin RPR in the Aspirin Group

The number of patients with high RPR according to PFA100 was 128 (25.5%). Patients with high RPR had significantly higher vWF levels (*P*<0.001) and platelet counts (*P*=0.05) (Table 3).

**Table 3.** Baseline Characteristics of the Aspirin Group (n=502) According to the Presence of High or Low RPR

	High RPR (n=128)	Low RPR (n=374)	P*
Age, y, mean (range)	61.9 (44.9–80.3)	62.1 (36.4–80.8)	0.85

Sex, female, n (%)	31 (24.2)	81 (21.7)	0.55

Race, white, n (%)	119 (93.0)	364 (97.3)	0.07

Cardiovascular risk factors, n (%)			

Current smoking	31 (24.2)	77 (20.6)	0.39

History of hypertension	63 (49.2)	209 (55.9)	0.19

Diabetes mellitus	29 (22.7)	76 (20.3)	0.58

Previous myocardial infarction	60 (46.9)	149 (39.8)	0.16

Intermittent claudication	5 (3.9)	24 (6.4)	0.29

Previous percutaneous coronary intervention	96 (75.0)	281 (75.1)	0.98

Previous coronary artery bypass grafting	26 (20.3)	77 (20.6)	0.95

Body mass index, kg/m^2^, median (25th, 75th percentiles)	26.9 (25.4, 29.3)	27.4 (24.9, 30.3)	0.52

Total cholesterol, mmol/L, mean±SD	4.46±0.89	4.60±0.99	0.14

High-density lipoprotein cholesterol, mmol/L, mean±SD	1.31±0.37	1.34±0.45	0.55

Low-density lipoprotein cholesterol, mmol/L, mean±SD	2.45±0.76	2.57±0.86	0.18

Triglycerides, mmol/L, median (25th, 75th percentiles)	1.32 (0.95, 1.90)	1.39 (0.93, 1.89)	0.61

Platelet count, ×10^9^/L, median (25th, 75th percentiles)	231 (203, 273)	224 (193, 264)	0.05

Mean platelet volume, fL, mean±SD	10.99±0.85	10.87±0.94	0.21

C-reactive protein, mg/L, median (25th, 75th percentiles)	1.85 (1.1, 3.4)	1.90 (0.9, 3.4)	0.33

vWF, %, median (25th, 75th percentiles)	125 (98, 145)	100(79, 125)	<0.001

TxB_2,_ ng/mL, mean (range)	2.9 (0.0–15.0)	2.6 (0.0–21.0)	0.32

Medication, n (%)			

Statins	128 (100)	367 (98.1)	0.15

β-Blockers	99 (77.3)	284 (75.9)	0.86

Calcium channel blockers	26 (20.3)	97 (25.9)	0.21

Angiotensin-converting enzyme inhibitors	31 (24.2)	90 (24.1)	0.93

Angiotensin receptor blockers	30 (23.4)	100 (26.7)	0.48

Proton pump inhibitors	13 (10.2)	44 (11.8)	0.62

Values are given as n (%), mean±SD, mean (range), median (25th, 75th percentiles), or percentage, as indicated.

* *P* for differences between groups.

Definitions as given in Table 1.

The associations between the presence of high RPR and clinical end points are shown in Table 4. The number of composite end points in the aspirin group was 54 (10.8%). High on-treatment RPR did not significantly influence the end point rate (17 of 128 [13.3%]) versus that obtained in patients with low RPR (37 of 374 [9.9%]) (*P*=0.31). There was also no statistically significant influence of high RPR on the different components of end points, although the proportion of patients who experienced a myocardial infarction was higher in patients with high RPR (6.3% versus 2.7%, *P*=0.07). vWF and platelet count were found to be effect modifiers. Therefore, separate analyses were undertaken with stratification on their respective median values. Patients with low vWF (≤106%) or low platelet count (≤227×10^9^/L) and high on-aspirin RPR had a significantly higher end point rate than that of patients with low on-aspirin RPR (20% versus 7.5%, OR 3.06, *P*=0.014, and 18.2% versus 10.8%, OR 2.25, *P*=0.039, respectively) (Table 5).

**Table 4.** Clinical Endpoints Among Aspirin Users (n=502) According to High or Low RPR as Determined by PFA100 and Among Patients With High On-Aspirin RPR (n=259) According to the Randomized Groups

	Aspirin Users (n=502)				Patients With High On-Aspirin RPR (n=259)					
	
Endpoints	No.	High RPR (n=128)	Low RPR (n=374)	Rate Ratio (95% CI)	*P*	No.	Aspirin (n=128)	Clopidogrel (n=131)	Rate Ratio (95% CI)	*P*
Composite	54	17 (13.3)	37 (9.9)	1.34 (0.76, 2.38)	0.31	27	17 (13.3)	10 (7.6)	1.74 (0.80–6.77)	0.16

Unstable angina	15	4 (3.1)	11 (1.9)	1.06 (0.34, 3.34)	0.92	8	4 (3.1)	4 (3.1)	1.02 (0.26–4.09)	0.97

Myocardial infarction	18	8 (6.3)	10 (2.7)	2.34 (0.92, 5.92)	0.07	12	8 (6.3)	4 (3.1)	2.05 (0.62–6.80)	0.23

Ischemic stroke	17	4 (3.1)	13 (3.5)	0.90 (0.29, 2.76)	0.85	6	4 (3.1)	2 (1.5)	2.05 (0.37–11.17)	0.39

Death	4	1 (0.8)	3 (0.8)	0.97 (0.10, 9.36)	0.98	1	1 (0.8)	0 (0.0)	Undefined	0.31

Person-years	…	256	748	…	…	…	256	262	…	…

Values are given as No. patients (%).

**Table 5.** Composite Endpoints Among Aspirin Users (n=502) According to High or Low RPR as Determined by PFA100 Based on Stratification Analyses

	Composite Endpoint		No Composite Endpoint					
						
	High RPR, n	Low RPR, n	High RPR, n	Low RPR, n	OR (95% CI)	*P*	OR_M-H (OR 95%)_	Homogeneity Across Strata
Total	17	37	111	337	1.39 (0.76–2.58)	0.29	…	…

vWF≤106%	8	16	32	196	3.06 (1.21–7.74)	0.014	…	…

vWF>106%	9	21	79	141	0.76 (0.33–1.75)	0.525	1.30 (0.71–2.40)	0.03

Platelets≤227	12	21	44	173	2.25 (1.03–4.91)	0.039	…	…

Platelets>227	5	16	67	164	0.76 (0.27–2.17)	0.614	1.45 (0.79–2.67)	0.11

OR_M-H_: indicates odds ratio (Mantel-Haenszel method).

Platelets: n×10^9^/L.

### High On-Aspirin RPR in the Clopidogrel Group

In the group randomized to clopidogrel (n=499), there was no significant difference in end point rate between patients with high and low on-aspirin RPR (10 of 131 [7.6%] versus 42 of 368 [11.4%], respectively; *P*=0.24).

### High RPR in Total Study Population

The composite end point rate in patients with high on-aspirin RPR treated with clopidogrel was not different from patients treated with aspirin (7.6 versus 13.3%, *P*=0.16) (Table 4).

### Low RPR in Total Study Population

In patients with low on-aspirin RPR at baseline (total n=741), there was no significant difference in end point rate between the aspirin group and the clopidogrel group (38 of 337 [11.3%] versus 41 of 325 [12.6%], respectively; *P*=0.64).

### Bleedings and Adverse Events

During the follow-up period, there were 130 bleeding episodes, 7 major and 123 minor. There was a significantly lower frequency of total bleedings in the aspirin group than in the clopidogrel group (10.2% versus 15.8%, *P*=0.008). This difference was due to lower minor bleedings in the aspirin group (9.8% versus 14.8%, *P*=0.002). No difference in major bleedings between the randomized groups was observed (0.4% versus 1.0%, *P*=0.25). There were no differences in major or minor bleedings among patients with high versus low on-aspirin RPR (0 [0%] versus 7 [0.9%], *P*=0.12, and 30 [11.6%] versus 93 [12.6%], *P*=0.68, respectively).

## Discussion

The results from the present study show that high on-aspirin RPR, as determined by the PFA100 method in patients with CAD continuing on aspirin, did not predict clinical outcome after 2 years of follow-up. Nevertheless, borderline significance was noted with regard to the risk for myocardial infarction in patients with high on-aspirin RPR.

The ASCET study is, to the best of our knowledge, the first prospective, randomized trial in which platelet function testing has been related to clinical events in patients on single-antiplatelet therapy with aspirin. Recent evidence has shown that aspirin given once daily does not provide stable 24-hour antiplatelet protection in all patients with CAD. ^6^ In the present study, all patients were tested in fasting condition 24 hours after intake of 160 mg aspirin, contributing to more uniform pharmacokinetics in the studied population.

During recent decades, morbidity and mortality rates in patients with CAD have decreased.^26,27^ Despite a relatively large study population (n=1001), the observed end point rate was less than half of that expected and upon which the power calculation was performed. This explains the negative overall results from the study.

Our main results are not in accordance with previous trials in which platelet function assessed by the PFA100 method predicted outcome after coronary angioplasty.^10,11,14^ Similarly, a meta-analysis of 20 trials, including 2930 patients, demonstrated an overall OR for clinical events to be 3.85 (95% CI, 3.08 to 4.80; *P*<0.001) in patients with high on-aspirin platelet reactivity as determined by PFA100 and other platelet function tests.^13^ The PFA100 method, which was the only “point-of-care” test available when our study was started, has some limitations. It is only partly dependent on platelet cyclooxygenase-1 (COX-1) activity, and the low COX-1 specificity might to some degree explain the diverging results in studies on aspirin nonresponsiveness.^4,28^ Nevertheless, in our study, all patients on aspirin had low serum TxB_2_ levels, indicating that their COX-1 pathway of platelet activation was largely inhibited.^29,30^ The high RPR seems therefore to depend more on platelet activation via other activation mechanisms. It has been reported that the PFA100 method has a lower predictive value than that of COX-1–specific tests (platelet aggregometry tests).^3^ The PFA100 method might be more relevant than the more specific tests, because it records the RPR in aspirin-treated patients while their COX-1 pathways are inhibited.^31^

The closure time with the PFA100 method is shown to be prolonged in patients with very low levels of vWF.^32^ No patients in our study had pathologically low levels; thus, any influence of low levels of vWF on the frequency of high on-aspirin RPR should be disregarded. Nevertheless, patients continuing on aspirin with vWF levels below median value (106%) and high on-aspirin RPR had a statistically significant higher end point rate than patients with low RPR. This has not been described previously, and a possible explanation might be that patients with high on-aspirin RPR despite their lower vWF levels have other, more dominant platelet-activating pathways that are not inhibited by aspirin.

High RPR has been associated with high platelet count.^5^ This is in line with our findings. Patients with high on-aspirin RPR despite below-median platelet count (227×10^9^/L) had a significantly increased end point rate when compared to patients with low on-aspirin RPR. This might be explained by increased platelet turnover and an increased fraction of circulating immature platelets, which also might increase the event risk.^5^

It should be pointed out that the findings of higher end point rate in patients with below-median values of vWF and platelet count are post hoc analyses not included in the primary aims of the study. These findings should therefore be interpreted carefully and might be hypothesis generating for further studies.

In patients randomized to clopidogrel (n=499), the end point rate did not differ from the group that continued aspirin. This is in accordance with the results from the Clopidogrel versus Aspirin in Patients at Risk of Ischaemic Events (CAPRIE) trial. Although this trial showed an overall benefit of clopidogrel treatment versus aspirin, patients entering the trial because of CAD did not benefit from clopidogrel treatment.^17^ The composite end point rate in patients with high on-aspirin RPR treated with clopidogrel was not different from patients treated with aspirin (7.6% versus 13.3, *P*=0.16). We could not demonstrate that these patients would benefit from changing aspirin treatment to clopidogrel treatment. The observed, not statistically significant reduction in end point rate (43%) might, however, support the suggested hypothesis that platelet activation mechanisms other than the COX-1 pathway might be more important for platelet activation in patients with high on-aspirin RPR.^31^

Patients’ compliance with aspirin therapy, as determined by serum TxB_2_ in all patients, was excellent. The median TxB_2_ level was 2.7 ng/mL. In patients not on aspirin, the levels are typically ≈200 to 300 ng/mL.^8,33^ Thus, the lack of response to aspirin cannot be explained by noncompliance.

The main limitation in our study was lack of statistical power. Less than half of the estimated number of events dramatically reduced the possibility for statistically significant effects on clinical outcome. The estimation of end point rate was based on data from similar populations available in 2002.^34^ The reports on low predictive values of the PFA100 method, when compared to COX-1–specific tests like platelet aggregometry, might be considered a limitation in the present trial, even though it could be argued that a COX-1–nonspecific test can be more clinically relevant for identifying the RPR in aspirin-treated patients.^3,31^ In addition, any influence of “high on-clopidogrel RPR” on end point rate has not been taken into consideration.

## Conclusions

Response to aspirin treatment evaluated with the PFA100 method did not influence the overall clinical outcome and did not identify a group responsive to clopidogrel in stable CAD patients on single-antiplatelet therapy with aspirin after a follow-up of 2 years. Post hoc subgroup analysis raised the possibility that on-aspirin RPR might be predictive in patients with low vWF or platelet count, but these findings will require confirmation in future trials.
